# COVID‐19 and the heart: An update for clinicians

**DOI:** 10.1002/clc.23406

**Published:** 2020-06-12

**Authors:** Ahmed Goha, Kenechukwu Mezue, Paul Edwards, Felix Nunura, Dainia Baugh, Ernest Madu

**Affiliations:** ^1^ Aga Khan University Hospital Nairobi Kenya; ^2^ Heart Institute of the Caribbean and HIC Heart Hospital Kingston Jamaica; ^3^ Department of Hospital Medicine Altru Health System Grand Forks North Dakota USA

**Keywords:** acute myocardial injury, COVID‐19, myocaritis, NSAID, RAAS

## Abstract

SARS‐CoV‐2, the cause of the COVID‐19 pandemic has significantly impacted cardiovascular healthcare. Patients with pre‐existing cardiovascular disease are at higher risk of morbidity and mortality. The virus may affect the heart directly and indirectly with clinical syndromes of acute myocardial injury, myocarditis, acute coronary syndromes, heart failure, arrhythmias, and venous thromboembolism. Some therapeutics under investigation for COVID‐19 may also have adverse cardiac effects. The involvement of the RAAS system in viral entry makes it pertinent to consider the effects of medications that modulate the system. Comprehensive knowledge of peculiar cardiovascular manifestations of COVID‐19 and the role of RAAS in the prognosis of COVID‐19 disease is needed for optimal patient management.

## 1 INTRODUCTION

The world is facing the challenge of the pandemic caused by the novel coronavirus, SARS‐CoV‐2, which results in a disease syndrome known as Coronavirus disease 2019 (COVID‐19). This disease started as an outbreak in Wuhan, China in December 2019 and at May 23, 2020, the virus had spread to 216 countries, areas and territories across the world with 5 103 006 confirmed cases and 333 401 deaths.^1^ On March 11, 2020, the World Health Organization (WHO) declared the disease a global pandemic.

The clinical spectrum of COVID‐19 appears to be wide, encompassing asymptomatic infection, mild upper respiratory tract illness and severe viral pneumonia with respiratory failure, systemic inflammatory syndrome and even death.^2^ The respiratory tract is the primary target for SARS‐CoV‐2 virus, however cardiovascular involvement has been documented in different studies and the heart is involved in 40% of patients dying from COVID‐19 disease.^3^

Cardiovascular complications of influenza and coronavirus infection, including myocarditis, acute myocardial infarction, and exacerbation of heart failure have been documented during previous epidemics with significant impact on both morbidity and mortality.^4^ Underlying myocarditis has been described with electrocardiographic changes, troponin elevation, and echocardiographic evidence of diastolic and systolic dysfunction. In previous coronavirus epidemics, adverse outcomes including hypotension, arrhythmia, and sudden cardiac death have been reported in patients with pre‐existing cardiovascular disease.^5^

Patients with pre‐existing comorbidities are thought to be at an increased risk of infection with SARS‐CoV2 and also tend to have worse clinical outcomes. Specifically, patients with cardiovascular disease, diabetes and hypertension are thought to have a high complication rate with mortality rate of 10.5% reported in cardiac patients and mortality rates of 7.3% and 6.0% for diabetes and hypertension patients, respectively.^6^

## 2 COVID 19 AND THE HEART: EPIDEMIOLOGY

### 2.1 Sex

Men are at higher risk in the COVID‐19 epidemic. They are admitted to hospital at higher rates and suffer higher degrees of morbidity and mortality. United States data from the COVID‐19 net dataset (May 9, 2020) found that men represented 52.9% of the hospitalized population as compared to 47.1% being women. A report of 5700 patients from a New York hospital system found that men represented 60.3% of the admitted patients. COVID‐19 mortality in this study was higher in men than in women at every age.^7^ The pathophysiology and significance of male predominance of COVID‐19 disease is uncertain. Further study is ongoing in this area.

### 2.2 Age

As age increases, so does the risk of developing severe COVID‐19 disease. Data from the CDC in the United States reveal that patients with COVID‐19 disease less than 19 years of age have a risk of hospitalization that is 2% to 3% compared to a risk of hospitalization that is greater than 31% in patients above the age of 85.^8^ Furthermore, no patients in this cohort less than 19 years of age required ICU care. In the age group 20 to 45 years the hospitalization rate was 2% to 4% and in the 75 to 84 year cohort the rate of hospitalization increased to 11% to 31%.^9^ A trend was noted for increasing mortality with age in the United States with case fatality rates of 0.1% to 0.2% in patients less than 44 years of age and 10.4% to 27.3% in patient 85 years or older.^9^ Recently there have been reports of a rare multi‐system inflammatory syndrome associated with COVID‐19 disease resembling Kawasaki disease in children.^10^ Much remains unclear about how commonly this occurs and what the risk factors may be.

### 2.3 Co‐morbidities

Patients with pre‐existing co‐morbidities are thought to be at an increased risk of infection with SARS‐CoV2 and tend to have worse clinical outcomes. Specifically, patients with cardiovascular disease, diabetes and hypertension are thought to have a high complication rate with mortality rate of 10.5% reported in cardiac patients and mortality rates of 7.3% and 6.0% for diabetes and hypertension patients, respectively.^6^ In a review of 1590 Chinese patients, hypertension, cardiovascular disease, cerebrovascular disease, diabetes, hepatis B infection, COPD, and chronic kidney disease were found to increase the risk of severe infections with COVID‐19 disease. In this study, when looking at a composite endpoint of ICU admission, mechanical ventilation and death, this end point was reached in 4.5% of patients with no co‐morbidities, 19.3% of patients with one co‐morbidity and 28.5% of patients with 2 or more co‐morbidities.^2^ This suggests increasing severity of disease as the number of co‐morbidities increase. Studies in the United States have suggested that obesity may also be a risk factor for severe COVID‐19 disease.^7^

### 2.4 Race/ethnicity

Experience from the United States and UK have suggested that race/ethnicity may play a role in the severity of COVID‐19 infection. Data from the CDC found that in patients hospitalized for COVID‐19 disease, black patients represented 33% of the COVID‐19 inpatient population while representing only 18% of the community in general, respectively.^11^ This suggests a disproportionate impact on the African American population. Indigenous Peoples have also been noted to be at increased risk from COVID‐19 Disease. In the United States, the rates of COVID‐19 cases have been documented to be four times as high on reservations when compared to the country as a whole.^12^

### 2.5 Socio‐economic factors

Aside from race and ethnicity other socio‐economic factors have been shown to worsen outcome with COVID‐19 disease. Nursing Homes and Assisted living facilities have proved to be areas of easy spread with high subsequent morbidity and mortality. Both inmates and correctional staff in US prisons have been documented to be at risk. Homeless shelters have also been identified as being at risk for COVID‐19 disease.^8^

## 3 PATHOPHYSIOLOGY OF COVID‐19 AND CARDIAC INVOLVEMENT

**FIGURE 1:**
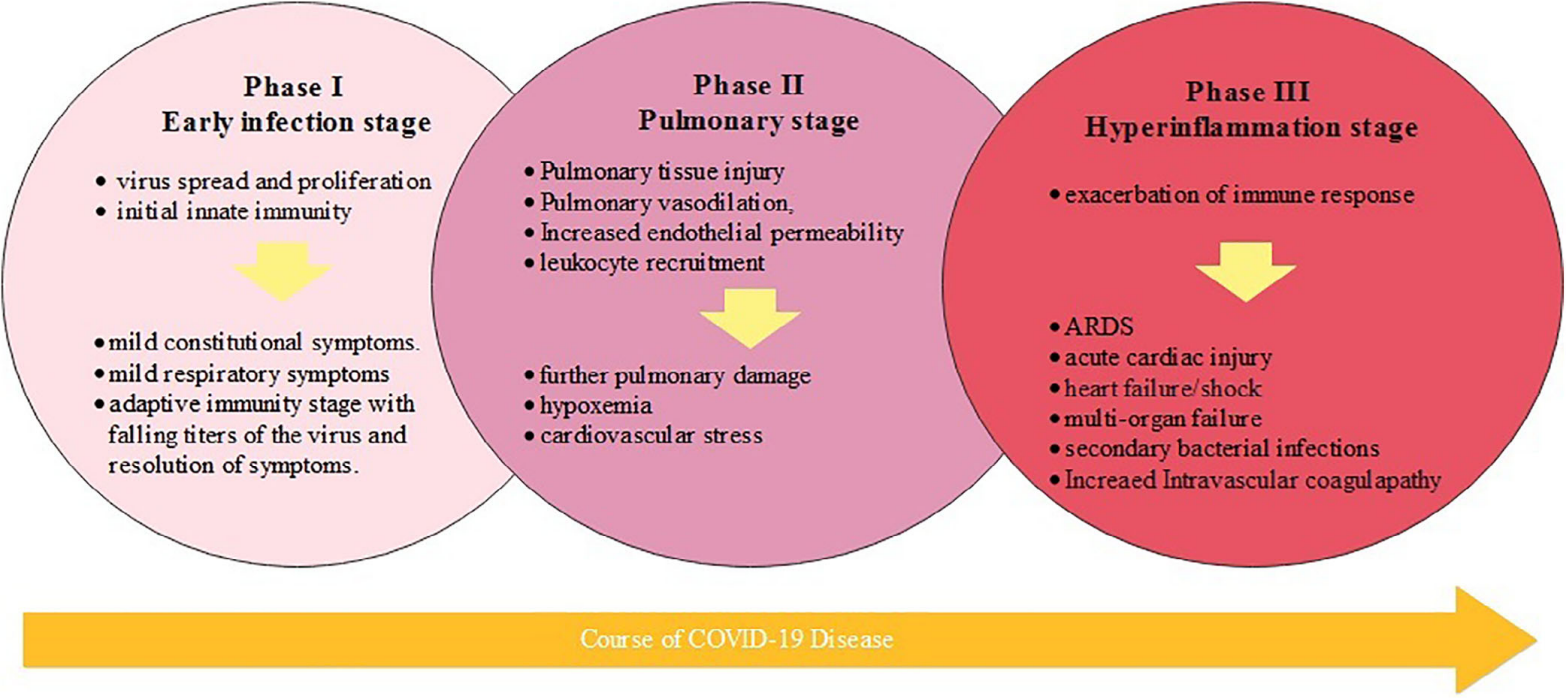
Progression of COVID‐19 infection

Progression of COVID‐19 disease is divided into three intermingling phases; early infection phase, a pulmonary phase, and a hyperinflammation phase^13^ (Figure 1). The first phase features virus spread and proliferation in lung tissues with initial innate immunity including recruitment of monocytes and macrophages, characterized by mild constitutional symptoms. Some early phase symptomatic patients suffer from mild respiratory symptoms and may require supportive care like supplemental oxygen followed by an adaptive immunity stage with falling titers of the virus and resolution of symptoms. The second stage includes several mechanisms leading to pulmonary tissue injury, vasodilation, endothelial permeability and leukocyte recruitment that cause further pulmonary damage, hypoxemia, and cardiovascular stress. Ten percent of patients in the second stage may experience further exacerbation of immune response (hyperinflammation stage) become critically‐ill, and they may suffer from acute respiratory distress syndrome (ARDS), acute cardiac injury, multi‐organ failure, secondary bacterial infections, sepsis and require intensive care .^13^

The mechanisms of cardiac injury are not well established. Acute myocardial injury is the most common cardiovascular complication in COVID‐19 manifested by elevation of high‐sensitivity cardiac troponin I and the incidence of acute myocardial injury has been reported to be around 8% to 12%.^14^ Acute myocardial injury has been shown to be a strong negative prognostic marker in patients with COVID‐19.^2^ Several mechanisms are thought to be responsible.:

*A. Direct myocardial injury*—SARS‐CoV‐2 viral invasion of cardiomyocytes is thought to occur by binding to angiotensin‐converting enzyme 2 (ACE2). The binding of SARS‐CoV‐2 to ACE2 can result in alteration of ACE2 signaling pathways, leading to acute myocardial and lung injury, however, autopsy studies have thus far failed to confirm this pathway.^15^ This will be discussed in detail in a later section of this article.

*B. Indirect myocardial injury*—which is expressed in two ways.

1. Overwhelming immune inflammatory response ‐ More severe forms of COVID‐19 disease are characterized by an acute systemic inflammatory response and cytokine storm, leading to multiple organ dysfunction syndrome (MODS). Several studies have documented high circulating levels of proinflammatory cytokines in patients with severe/critical COVID‐19 disease.^16^
2. Severe hypoxia from acute respiratory damage which may result in oxidative stress and increased cardiometabolic demand that leads to acute myocardial injury.^15^

### 3.1 Histopathology of COVID‐19 and cardiac involvement

There are only few postmortem studies, however, histopathologic changes are consistent with diffuse alveolar damage, reactive type II pneumocyte hyperplasia, hyaline membrane formation, intra‐alveolar fibrin exudates, epithelial damage, along with loose interstitial fibrosis and chronic inflammatory infiltrates.^17^ Intravascular coagulopathy was noted in an autopsy study with fibrin thrombosis of small arterial vessels in 87% of specimens. Microvascular thrombosis and hemorrhage linked to extensive alveolar and interstitial inflammation that shares features with the macrophage activation syndrome. This may explain the severe hypoxemia which characterizes the clinical feature of ARDS in COVID‐19 patients.^18^ Limited cardiac autopsies have shown some interstitial mononuclear inflammatory infiltrates, but no virus has been identified in cardiac tissues.^17^

### 3.2 Biomarkers of COVID‐19 and cardiac involvement

Laboratory results for patients with COVID 19 exhibit elevated cardiac biomarkers. Mild elevation of cardiac troponin levels (less than 2 to 3 times of upper limits of normal) have been observed and thought likely to be due to possible pre‐existing cardiac condition and/or acute injury related to COVID‐19. In patients with severe and fatal COVID‐19, higher levels of cardiac troponin (more than five times upper limits of normal) suggests severe respiratory failure, tachycardia, systemic hypoxemia, myocardial injury either from potential direct or indirect viral myocarditis, endothelial dysfunction or plaque rupture triggered by COVID‐19 infection with subsequent acute coronary syndrome, Takotsubo syndrome or progression to multiple organ failure.^19^

Severe inflammatory and/or respiratory illnesses is usually accompanied by elevated BNP/NT‐proBNP. High BNP/NT‐proBNP levels correlates with the extent of right ventricular stress. Elevations of D‐Dimers have been associated with poor outcome. D‐dimer on admission greater than 2.0 μg/mL (fourfolds) correlates with COVID‐19 patients in‐hospital mortality.^20^

## 4 PECULIAR CARDIAC‐RELATED MANIFESTATIONS OF COVID‐19

### 4.1 Myocarditis

**FIGURE 2:**
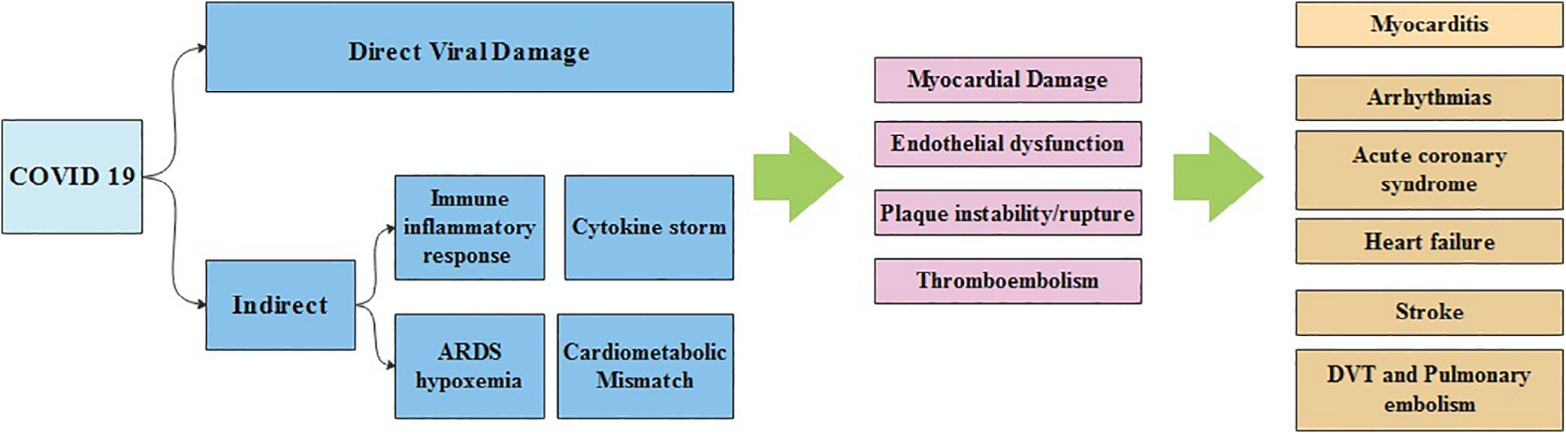
Pathophysiology of COVID‐19 cardiac related complications

The actual incidence of myocarditis with COVID‐19 infection is undetermined, however it is assumed that up to 7% of the COVID‐19‐related deaths are linked to myocarditis. The spectrum of symptoms may vary from mild symptoms such as mild chest pain, dyspnea and fatigue to more severe symptoms with left and right ventricular failure, cardiogenic shock, arrythmia, and sudden cardiac death with fulminant myocarditis.^21^ Although there is no strong evidence supporting direct COVID‐19 viral myocarditis so far, the viral RNAs of MERS‐CoV and SARS‐CoV, which are close relatives of SARS‐CoV‐2 have been found in the heart tissues of infected animals. COVID‐19 myocarditis is likely due to a combination of direct cell injury and T‐lymphocytes‐mediated cytotoxicity which can be augmented by the cytokine storm syndrome.^21^ COVID‐19 induced myocarditis may mimic an acute coronary syndrome with ST segment elevation and elevated enzymes due to acute cardiac injury and hence, providers should be aware of this presentation^22^ (Figure 2).

### 4.2 Arrhythmias

The most commonly reported arrhythmia in COVID‐19 disease is symptomatic/asymptomatic tachycardia. Bradycardia has been also reported. Arrythmia may occur in the setting of myocarditis, myocardial ischemia and in critically ill patients with hypoxia and shock.^23^ Other forms of arrythmias have been reported. Several mechanisms may trigger or aggravate arrhythmias in subjects with COVID 19. Potential causes include electrolyte disturbance (mainly hypokalemia), adverse effects of therapies (eg, chloroquine/hydroxychloroquine and azithromycin) that prolong QT interval with potential development of polymorphic ventricular tachycardia (VT)^24^ and fever which may unmask cases of cardiac channelopathies such as Brugada syndrome and long QT syndrome.^6^

### 4.3 Acute coronary syndrome

There are no clear statistics on the incidence of ST segment elevation myocardial infarction (STEMI) from intracoronary plaque rupture or obstruction in the setting of COVID 19 disease and it is likely low. Acute coronary events may be triggered by plaque rupture and coronary thrombosis due to inflammation/increased shear stress in high risk patients. Tam et al, reported a significant decline in the number of patients with STEMI seeking medical care at their institute.^25^ They attributed this to the reluctance of patients to go to a hospital during the COVID‐19 outbreak, delays in evaluating patients with STEMI after hospital arrival due to precautions such as detailed travel and contact history, symptomatology, and chest X‐ray. Taken together, this may delay patient transfer to catheterization laboratory. Additional precautions taken in catheterization laboratory such as time needed to wear protective gear may further delay intervention.^25^

### 4.4 Heart failure

There is a lack of data on the incidence of left ventricular systolic dysfunction, acute left ventricular failure, and cardiogenic shock. One study demonstrated heart failure in 52% of deceased patients and 12% of discharged patients.^2^ Many critically ill patients may develop reversible sepsis‐related cardiomyopathy with left ventricular dilatation, impaired systolic function and recovery within 7 to 10 days.^21^ COVID‐19 infection can cause decompensation of underlying heart failure and may lead to mixed shock syndrome (combination of septic shock and cardiogenic shock). Invasive hemodynamic monitoring, if feasible, may be helpful to manage the cardiac component of shock in such cases.^26^

### 4.5 Long‐term cardiovascular outcome

It is too early to predict long term cardiovascular outcome for patients who have recovered from COVID 19 infection, However, the potential outcome may be similar to that seen in the severe acute respiratory syndrome (SARS) caused by the SARS‐CoV virus. The outcome studies of patients who recovered from SARS and were followed for 12 years showed that 40% had cardiovascular abnormalities, 60% with altered glucose metabolism and 68% with abnormal lipid metabolism.^25^

### 4.6 Cerebrovascular disease

COVID‐19 infection is associated with a prothrombotic state causing venous and arterial thrombosis and elevated D‐dimer. The reported incidence of cerebrovascular disease among severe COVID‐19 patients varied from 2.3% to 22%.^27^Increased production of antiphospholipid antibodies has been suggested as a possible cause of ischemic stroke.^28^ Stroke has been found to be associated with a ∼2.5‐fold increased disease severity in patients with COVID‐19.^27^

### 4.7 Hemostasis and thrombosis

Laboratory and autopsy results have found evidence of a hypercoagulable state with severe COVID‐19 infection. Coagulation factors and platelets are involved in modulation of the host immune response, displaying proinflammatory functions that are independent from their hemostatic effects. Elevated D‐dimer has been linked to worse outcome.^20^

## 5 THERAPEUTIC OPTIONS IN COVID TREATMENT: CARDIAC IMPLICATIONS

There are various medications (new and established) that are currently being tested in randomized clinical trials (RCT) for the treatment of COVID‐19 disease. Chloroquine (an old antimalarial medication), and hydroxychloroquine (used in treating some rheumatologic conditions) showed good in‐vitro activity against the virus and there was great excitement about the potential of these medications in the early stages of the pandemic.^29^ However, the RCTs so far have not shown any benefit for chloroquine or hydroxychloroquine, rather, a study had to be stopped mid‐trial due to side effects of therapy as 11 patients died in the group taking chloroquine. In an analysis of a multinational registry of 96 032 patients from 671 hospitals in six continents, Mehra and colleagues^30^ found no benefit of Hydroxychloroquine or chloroquine, when used alone or with a macrolide, on in‐hospital outcome for COVID‐19. Each of these drug regimens was associated with decreased in‐hospital survival and an increased frequency of ventricular arrhythmias when used for treatment of COVID‐19. Complications of the drug include severe cardiac electrical abnormalities electrical irregularities in the heart such as polymorphic VT (Torsade de Pointes), long QT syndrome, and increased risk of sudden death.^19^ Remdesivir, a nucleotide analogue prodrug that inhibits viral RNA polymerases and which was initially developed to treat the Ebola virus has also shown good in‐vitro activity against the SARS‐CoV‐2 virus.^29^ Clinical trials on Remdesivir are currently ongoing and two trials have reported a reduction in time to clinical improvement in patients who took the drug compared to placebo.^31^ Convalescent plasma and tocilizumab (a monoclonal antibody against interleukin‐6) are other therapies that have shown some promise in reducing severity of illness in case series but the results of RCTs on these are still awaited.^32^

## 6 COVID‐19 AND THE RAAS SYSTEM

The renin angiotensin aldosterone system (RAAS) is a complex hormonal system that is comprised of the interactions of renin, angiotensinogen, angiotensin converting enzyme (ACE), angiotensin converting enzyme 2 (ACE2), angiotensin II, and aldosterone. In summary, RAAS is a system that responds to a physiologic state of hypovolemia, hyponatremia, adrenergic activation and hypotension and activates a system that increases vasoconstriction and fluid retention.^33^ ACE2 plays a role as a negative regulator in this system. The spike (S) protein of SARS‐CoV‐2 enables the virus to enter into the host cells by binding to ACE2 protein on the cell membrane. This is similar to the mode of entry of the causative agent of severe acute respiratory syndrome, SARS‐CoV which caused an epidemic in 2002/2003. The ACE2 receptor is found in the cells of the upper respiratory tract and the alveoli of the lung and is the primary site of entry of the virus in the body.^34^ It is also found in other tissues in varying amounts including the gastrointestinal tract (which is probably the reason for a common presenting symptom of diarrhea) and heart muscle (which might explain the cardiac manifestations of COVID19 disease).

The involvement of the RAAS system in viral entry makes it pertinent to consider the effects of medications that modulate the system. Angiotensin converting enzyme inhibitors (ACEi) and angiotensin receptor blockers (ARBs) are common medications that are used to treat hypertension. In a large study of a cohort of COVID‐19 patients, it was shown that there is an excess prevalence of hypertension in severe cases with poor outcomes suggesting that these medications might play a role in the pathology of the infection.^3^ In that cohort of 1099 patients, 165 [15%] of those patients had hypertension, but 24 [35.8%] of the 67 with poor outcomes (defined as admission to an intensive care unit, the use of mechanical ventilation or death) were hypertensive.

ACEi and ARB cause upregulation of ACE2 and this is thought to increase viral entry .^35^ On the other hand, markedly elevated angiotensin II levels were noted in plasma samples from SARS‐CoV‐2 infected patients and were linearly associated with viral load and lung injury. Indeed, enhanced ACE activity and decreased ACE2 activity have been shown to contribute to lung injury in mouse models^33^ .This suggests that medications like ACEi and ARBs which decrease ACE activity and increase upregulation of ACE2 might have beneficial effects.

Therefore, from a theoretical standpoint, ACEi and ARBs might facilitate viral entry into respiratory cells leading to viral mediated cell damage, but on the other hand, these same medications might upregulate ACE2 and ameliorate the acute lung injury caused by the virus. It is not clear which of these activities are predominant in individuals with COVID‐19 and further research is needed. In the meantime, most current cardiology guidelines recommend that patients on ACEi and ARBs should continue taking their medications as usual an should not discontinue them with CoVID‐19 disease.^36^

## 7 STATINS AND COVID‐19

Statins are effective cholesterol lowering drugs that act primarily by blocking the 3‐hydroxy‐3‐methyl‐glutaryl‐CoA (HMG‐CoA) reductase enzyme. It was found that statins, particularly pitavastatin, bind strongly to and inhibit the SARS‐CoV‐2 main protease (Mpro), a key coronavirus enzyme, which is a potential drug target.^37^ This will need to be explored in further studies.

## 8 COVID‐19 AND NSAIDS

Non‐steroidal anti‐inflammatory drugs (NSAIDs) are medications that work by inhibiting the cyclo‐oxygenase enzyme that produces prostaglandins and are commonly used for the relief of pain and fever. Initial public concern for these medications in the context of COVID‐19 was raised by the French Minister of Health in mid‐March and the basis of his concern appeared to be an anecdotal report of four patients who had appeared to worsen in the context of NSAID use. These medications have been shown to increase the expression of ACE2 on the cellular membranes and could theoretically increase viral entry into respiratory cells.^38^ However, aside from case reports, there is no other evidence that NSAIDs lead to poor outcomes in COVID‐19 patients and in contrast, one NSAID, Indomethacin has shown in vitro evidence of inhibition of RNA synthesis of the SARS‐CoV‐1 virus independent of its cyclooxygenase activity.^39^ More studies will be needed on this, but given this concern, many European countries have suggested that in patients with respiratory tract infections, acetaminophen should be used in preference to NSAIDs for control of pain and fever.^40^

## 9 CONCLUSION

This review has examined the cardiac complications of COVID‐19 and also sheds a light on peculiar characteristics of the virus and its relationship with cardiac therapies particularly ACEi and ARBs. More research is ongoing on various aspects of this novel coronavirus and clinicians are watching keenly to learn as much as they can in the process.

## CONFLICT OF INTEREST

The authors declare no potential conflict of interests.
